# Fashion braces: an alarming trend

**DOI:** 10.1590/1516-3180.2018.0296250718

**Published:** 2018-09-06

**Authors:** Shahryar Sorooshian, Alangseri Ayubayu Kamarozaman

**Affiliations:** I PhD. Associate Professor, Faculty of Industrial Management, Universiti Malaysia Pahang Ringgold Standard Institution, Gambang Kuantan, Pahang, Malaysia.; II MBA Student, Faculty of Industrial Management, Universiti Malaysia Pahang Ringgold Standard Institution, Gambang Kuantan, Pahang, Malaysia.

Dear Editor,

Fixed orthodontic appliances, also known as braces, are apparatuses used for orthodontic treatment and are commonly used among teenagers. Certified orthodontists use braces to move unaligned teeth until they become well-arranged.

In Southeast Asian countries, the ratio of orthodontists to populace is much less than in many other countries; for instance, in Malaysia it is 1:220,000.^1^ The limited number of specialists contributes towards inability to satisfy the demand. Thus, orthodontic treatment has become an expensive service in non-government clinics. Although the cost is less through government services, the waiting list is very long and most often only the patients with severe disorders will be selected. Hence, the high cost of treatments using braces confers luxury prestige to this apparatus. Moreover, use of braces is renowned for its connotation of spending power and therefore constitutes an alluring status symbol among teenagers across Southeast Asia.^2^ This has resulted in a new trend for fashion braces, which are traded through the black market.

Fashion braces, also known as fake braces, look very comparable to real orthodontic braces but are not operative. They do not have any therapeutic purpose, but the black market for fashion braces targets teenagers who are seeking to attain the standard of luxury denoted by their use. Fashion braces can be supplied with a variety of ornamentation such as diamonds or cartoon characters attached to colorful orthodontic rubber bands.

Fashion braces are sold over the counter on the streets and online. Some websites and social-media platforms even provide do-it-yourself (DIY) videos. In some Southeast Asian countries, fake brace providers are mostly individuals who do not have orthodontic certification or qualification. They are not legally permitted to conduct dentistry practice and so they cannot register their offices. Without legal premises, they will usually arrange their customers’ appointments in beauty salons^3^ or at residential addresses.

These braces are affixed without proper clinical assessment and without any radiographic investigation prior to use. The availability of clinical apparatus and its hygiene status may be questionable especially in relation to DIY brace-fixing practices. Patients do not have any right to reclamation in the event of an injury, infection or any other issue during or after installation of the braces.

This substandard orthodontic practice can cause damage to patients’ teeth, gums, lips and supporting bones. The authorities claim that these practices may also cause internal damage and cancer.^4^ Moreover, there have been cases of teenagers who died after using fashion braces.^5^ In the news this year (2018), some fashion braces have been found to present inferior material quality and have been laden with toxic heavy metals such as cadmium.^5^

The fashion-brace trend started in Southeast Asia and then expanded to the Far East. Recently, the trend reached the Middle East and it is still growing towards other parts of the world. Hence, it can be suggested that enforcement of laws and policies needs to be strengthened to monitor and prevent the use of fake braces. Higher authorities need to look at this issue seriously. Society needs to be made aware of the negative impact of fake braces, especially in relation to teenagers.
